# Quantitative pulmonary blood flow measurement using ^15^O-H_2_O PET with and without tissue fraction correction: a comparison study

**DOI:** 10.1186/s13550-017-0350-8

**Published:** 2017-12-22

**Authors:** Keiko Matsunaga, Masahiro Yanagawa, Tomoyuki Otsuka, Haruhiko Hirata, Takashi Kijima, Atsushi Kumanogoh, Noriyuki Tomiyama, Eku Shimosegawa, Jun Hatazawa

**Affiliations:** 1 Department of Molecular Imaging in Medicine, Osaka University Graduate School of Medicine, Suita, Osaka, Japan; 2 Department of Nuclear Medicine and Tracer Kinetics, Osaka University Graduate School of Medicine, 2-2 Yamadaoka Suita city, Osaka, Japan; 3 Diagnostic and Interventional Radiology, Osaka University Graduate School of Medicine, Suita, Osaka, Japan; 4 Department of Respiratory Medicine, Allergy and Rheumatic Disease, Osaka University Graduate School of Medicine, Suita, Osaka, Japan

**Keywords:** PET, ^15^O-H_2_O, Pulmonary blood flow, Tissue fraction correction

## Abstract

**Background:**

Physiological measures per lung parenchyma, rather than per lung volume, sometimes reflect the disease status. PET images of the lung, which are usually expressed per lung volume, could confound the interpretation of the disease status, especially in cases with a prominent heterogeneity in aeration. The aim of the present study was to develop a method for measuring pulmonary blood flow (PBF) with aeration correction using ^15^O-H_2_O PET and to compare the results with those obtained using a conventional method. We obtained the voxel-based tissue fraction (TF) derived from density images converted from transmission images obtained using an external ^137^Cs point source. Quantitative PBF values with and without the TF were calculated using ^15^O-H_2_O PET to examine contralateral lung tissue in 9 patients with unilateral lung cancer. The heterogeneity in PBF before and after TF correction was then evaluated and compared. As a measure of PBF heterogeneity, we used the skewness and kurtosis of the PBF distribution.

**Results:**

The mean PBF of contralateral lung was 1.4 ± 0.3 mL/min per mL of lung. The TF-corrected PBF was 5.0 ± 0.6 mL/min per mL of lung parenchyma. After TF correction, the skewness and kurtosis of the PBF decreased significantly.

**Conclusions:**

The present PBF calculation method using TF correction demonstrated that the normal PBF increased significantly and the PBF distribution became uniform. The proposed TF correction method is a promising technique to account for variations in density when interpreting PBF in PET studies.

## Background

The lung is a unique organ comprised of some tissue and a large quantity of air. The average size of human lung alveoli is much smaller than the usual PET voxel size [1], making it difficult to measure physiological values per lung parenchyma using PET. Physiological values per parenchyma can be obtained using the volume fraction of parenchyma per lung, to which we refer hereafter as the tissue fraction (TF) [2, 3]. TF heterogeneity is prominent in patients with chronic obstructive pulmonary disease (COPD) or interstitial lung disease (ILD) [4, 5]. Recently, Lambrou et al. [2] and Holman et al. [3] presented a method for calculating the standardized uptake value (SUV) and kinetic parameters in lung with TF correction using the density distribution derived from CT. They applied this method to ^18^F-FDG SUV images in patients with ILD. Before correction, the ^18^F-FDG uptake seemed to be restricted to the CT-identified fibrotic region. After TF correction, however, the ^18^F-FDG uptake increased not only in the fibrotic region, but also in normal-appearing lung regions. In addition, the ^18^F-FDG uptake in normal-appearing lung tissue in patients with ILD was significantly higher than the uptake in normal subjects, suggesting that normal-appearing lung tissue is also affected by the disease process. TF-corrected images could clarify the disease process in the lung.

^15^O-H_2_O PET using a one-tissue compartment model [6, 7] is the reference standard for measuring pulmonary blood flow (PBF) per lung volume, and this technique has been validated against ^68^Ga microsphere measurements in dogs [8] and ^68^Ga-labeled macroaggregate measurements in humans [9]. However, the PBF per lung parenchyma with TF correction using ^15^O-H_2_O PET has not yet been reported.

The TF distribution is derived from the density distribution, which can be obtained from transmission images using CT or an external source. Rhodes et al. [10] and Schuster et al. [11] reported that density was linearly correlated with the attenuation coefficient derived from a 15-min ^68^Ga/^68^Ge transmission scan. Transmission data reflects the time-averaged density distribution over several respiratory cycles, which can be easily registered with PET data. Therefore, in PET scanners equipped with an external radioactive source, TF-corrected images can be easily obtained using transmission data.

In pulmonary kinetic PET studies with TF correction, the fraction of the blood volume in the lung should be considered, since 20 % of the lung volume is blood [10]. However, a method for measuring PBF with correction for the pulmonary blood volume has not been fully established.

The aim of the present study was to develop a method for measuring PBF per parenchyma using the TF derived from transmission images. We applied this method to contralateral lung tissue in patients with lung cancer and examined the heterogeneity in PBF before and after TF correction. We also investigated the effect of blood volume correction on PBF measurements.

## Methods

### Subjects

Between April 2012 and July 2015, 13 patients with stage 4 non-small cell lung cancer, who were scheduled to receive chemotherapy at the Osaka University Hospital, underwent ^15^O-H_2_O dynamic PET. All the patient scans were obtained as part of a prospective study designed to evaluate lung cancer and PBF before and after the administration of bevacizumab. The permission of the Institutional Review Board and informed patient consent were obtained. To analyze normal lung, 9 patients (6 men, 3 women; mean age ± standard deviation (SD), 60 ± 10) with unilateral lung cancer were chosen. We analyzed the PBF of the contralateral lung. The patient characteristics are summarized in Table 1.

**Table 1:** Patient characteristics

Patient no.	Sex	Age (years)	Lesion	Smoker	Pack-years	FEV1/FVC (%)	LAA_−950_ (%)
1	M	62	Right	Current-smoker	83	NA	7.7
2	F	61	Left	Ex-smoker	10	73.2	8.4
3	M	62	Right	Current-smoker	80	70.3	12.2
4	M	46	Right	Ex-smoker	24	NA	12.2
5	M	61	Right	Ex-smoker	80	NA	10.5
6	M	64	Right	Ex-smoker	80	62.6	5.4
7	F	42	Right	Ex-smoker	33	70.3	3.0
8	F	73	Right	Never-smoker	0	NA	5.9
9	M	71	Left	Current-smoker	51	50.2	25.9

*NA* not available

### CT analysis to examine the emphysematous change

Eight of nine patients in this study had a history of smoking, which is a risk factor of COPD [12]. Emphysema is sometimes associated with COPD, which can cause TF heterogeneity. The relative area of lung parenchyma with attenuation coefficients of less than −950 HU on thin-section CT scans obtained during inspiration is reported to be an objective measurement of the extent of macroscopic emphysema and a reflection of microscopic emphysema [13]. We examined non-contrast chest high resolution (HR) CT of each patient to examine the presence of emphysematous change. In eight of nine cases, HRCT was taken within 1 year before or after ^15^O-H_2_O PET. In one case (No. 2), non-contrast HRCT was taken 1.5 years before ^15^O-H_2_O PET. Images were acquired in the supine position with a 0.625 mm slice thickness, a 0.4 s rotation time, a beam pitch of 0.98, and 120 kV of x-ray tube voltage. The tube current was determined by automatic exposure control, which is clinically used for dose reduction. During scanning the patients were asked to hold their breath after a deep inspiration.

We used lung analysis module of Fujifilm Synapse Vincent system (Fujifilm Corporation, Tokyo, Japan) to obtain the volume fraction of lung parenchyma with attenuation coefficient of less than −950 HU to the whole contralateral lung (LAA_-950_).

### Spirometry

Five patients (No. 2, 3, 6, 7, 9) underwent spirometry within 1 year of PET. We examined the ratio of forced expiratory volume in 1 s to forced vital capacity (FEV1/FVC) as indication of airflow limitation, which is accompanied with COPD.

### Phantom study to investigate the relationship between density and the linear attenuation coefficient

Prior to applying our proposed technique to the patient datasets, we performed phantom studies to confirm the linear relationship between the linear attenuation coefficient derived from the transmission scan and density. Studies were performed using a SET-3000 GCT/X scanner (Shimadzu Corp., Kyoto, Japan). The transmission scanner had an axial length of 23 mm and was equipped with a rotating ^137^Cs point source and a tungsten collimator. The rotation speed was 3 s per cycle [14]. We performed 5-min transmission scans. The transmission image shows the distribution of the linear attenuation coefficient (cm^−1^), which was reconstructed using a maximum a posteriori reconstruction.

Following the methods reported by Rhodes et al. [10] and Schuster et al. [11], we performed transmission scans using a variety of phantoms with known densities (in g/mL): Styrofoam (0.016), sawdust (0.11), Japanese cypress (0.40), ethanol for disinfection (0.86), cat litter containing of minerals (0.89), and water (1.00). The sawdust, ethanol, cat litter, and water were placed within a light polyethylene container of known weight and volume and scanned. The densities of these materials were then plotted against the attenuation coefficient obtained by the transmission scan.

### Theory

#### Derivation of TF

We assumed that a lung voxel would contain three components: air, parenchyma, and blood [3]. *ρ*_*lung*_ (see Appendix for list of variables) can be considered as

1 $$ {\rho}_{lung}={\rho}_{air}\cdot {V}_{air}+{\rho}_{pa}\cdot {V}_{pa}+{\rho}_v\cdot {V}_V. $$

By definition, *V*_*air*_ + *V*_*pa*_ + *V*_*V*_ equals 1. *ρ*_*pa*_ and *ρ*_*v*_ are approximately equivalent to the averaged density of the mediastinal blood pool *ρ*_*med*_ (g/cm^3^). Since *ρ*_*air*_ (1.2 × 10^−3^ g/cm^3^) is about 1000 times smaller than *ρ*_*med*_ (1.06 g/cm^3^), *ρ*_*air*_ ⋅ *V*_*air*_ is negligible in Eq. 1.

Therefore,

2 $$ {\displaystyle \begin{array}{ll}{\rho}_{lung}& ={\rho}_{med}\cdot {V}_{pa}+{\rho}_{med}\cdot {V}_V\\ {}& ={\rho}_{med}\left(1-{V}_{air}\right)\end{array}} $$

We obtained *V*_*air*_ as follows:

3 $$ {V}_{air}=1-{\rho}_{lung}/{\rho}_{med}. $$

If density and the attenuation coefficient derived from the transmission scan have a linear relationship, Eq. 3 is expressed as,

4 $$ {V}_{air}=1-{T}_{lung}/{T}_{med}. $$

*T*_*lung*_/*T*_*med*_ corresponds to TF.

*C*_*P*ET_ can be considered as,

5 $$ {\displaystyle \begin{array}{ll}{C}_{PET}& ={V}_{air}\cdot {C}_{PET_{air}}+{V}_{pa}\cdot {C}_{PET_{pa}}+{V}_{\mathrm{v}}\cdot {C}_{RV}\\ {}& =\left(1-{V}_{air}-{V}_V\right)\cdot {C}_{PET_{pa}}+{V}_{\mathrm{v}}\cdot {C}_{RV},\end{array}} $$

where ${C}_{PET_{air}}$, ${C}_{PET_{pa}}$, and *C*_*RV*_ are the radioactivities of air, lung parenchyma, and pulmonary circulation blood. ${C}_{PET_{air}}$ equals 0.

#### PBF with and without TF correction

We used the standard single-tissue-compartment model [8] to calculate the PBF without TF correction as follows:

6 $$ {C}_{PET}(t)=F\cdot {C}_{RV}(t)\otimes {e}^{-\left(\frac{F}{V_T}+\lambda \right)t}, $$

where, ⊗ is the convolution operation.

${C}_{PET_{pa}}$ would be represented as follows using PBF with TF *F*_*corr*_:

7 $$ {C}_{PET_{pa}}={F}_{corr}\cdot {C}_{RV}(t)\otimes {e}^{-\left(\frac{F_{corr}}{V_{Tcorr}}+\lambda \right)t}. $$

Thus, the relationship between *C*_*PET*_ and *F*_*corr*_, *V*_*Tcorr*_ was as follows:

8 $$ {\displaystyle \begin{array}{l}{C}_{PET}=\left(1-{V}_{air}-{V}_V\right)\cdot {F}_{corr}\cdot {C}_{RV}(t)\otimes {e}^{-\left(\frac{F_{corr}}{V_{Tcorr}}+\lambda \right)t}+{V}_{\mathrm{v}}\cdot {C}_{RV}\\ {}=\left({T}_{lung}/{T}_{med}-{V}_V\right)\cdot {F}_{corr}\cdot {C}_{RV}(t)\otimes {e}^{-\left(\frac{F_{corr}}{V_{Tcorr}}+\lambda \right)t}+{V}_{\mathrm{V}}\cdot {C}_{RV}.\end{array}} $$

We also assessed a model with TF correction but without pulmonary circulation blood volume correction:

9 $$ {C}_{PET}=\left(1-{V}_{air}\right)\cdot {F}_{corr\hbox{'}}\cdot {C}_{RV}(t)\otimes {e}^{-\left(\frac{F_{corr\hbox{'}}}{V_{Tcorr\hbox{'}}}+\lambda \right)t}. $$

**Fig. 1:**
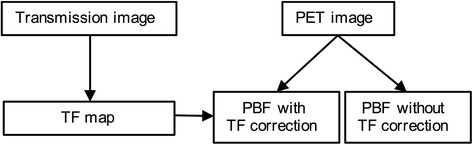
Flow chart of the algorithm

A flow chart of the analytical procedures is shown in Fig. 1.

The need to include a correction for the pulmonary circulation blood volume in the model was assessed using the Akaike information criteria (AIC) [15].

### Scanning protocol

Studies were performed using a SET-3000 GCT/X scanner (Shimadzu Corp., Kyoto, Japan). This scanner has an axial field of view of 26 cm, divided into 99 contiguous planes. The intrinsic spatial resolution is 3.5 mm full width at half maximum (FWHM) in-plane and 4.2 mm FWHM axially [14]. Patients were positioned supine in the scanner bed, with both the tumor and aortic arch or heart in the center of the axial field of view. For attenuation correction, a transmission scan (5 min) was performed using a ^137^Cs point source. Transmission images were also used for the derivation of TF images. After the transmission scan, a 10-min list mode scan was started simultaneously with an intravenous injection of 185 MBq of ^15^O–H_2_O (18.5 at 0.5 mL/s). The images were corrected for scatter radiation using the hybrid dual-energy window method [16]. The emission scan was reconstructed into 22 frames (1 × 10, 8 × 5, 4 × 10, 2 × 15, 3 × 20, 2 × 30, and 6 frames × 60 s) using the two-dimensional dynamic row-action maximum-likelihood algorithm (DRAMA) after 3-D Gaussian smoothing with a 6-mm FWHM. The voxel size was 4.7 × 4.7 × 2.6 mm.

### Input function

The volumes of interest (VOIs) were drawn over the right ventricular cavity manually so as to include the hottest voxel in approximately five consecutive image planes of the frame in which the first pass of the bolus was best visualized. The average VOI was 2.3 cm^3^. The projection of the resulting VOI onto all the image frames yielded the time–activity curve for the pulmonary circulation *C*_*RV*_ (t).

### Parametric imaging

Parametric PBF images with and without TF correction were generated using the basis function method [17].

Equation 8, which is used to obtain PBF with TF, is rewritten as follows:

10 $$ {C}_{PET}(t)=\left({T}_{lung}/{T}_{med}-{V}_V\right)\cdot {F}_{corr}\cdot {B}_{corr}+{V}_V\cdot {C}_{RV}, $$

where *B*_*corr*_ = *C*_*RV*_(*t*) ⊗ *e* ^−*θt*^ and *θ* = *F*_*corr*_/*V*_*Tcorr*_ + *λ*. The nonlinear term *B*_*corr*_ in Eq. 10 including *θ* is precalculated as the discrete basis function *B*_*corri*_ for the available range of *θ*:

11 $$ {B}_{corri}=\kern0.5em {C}_{RV}(t)\otimes {e}^{-{\theta}_it}. $$

Using the basis function *B*_*corri*_, Eq. 10 becomes:

12 $$ {C}_{PET}(t)=\left({T}_{lung}/{T}_{med}-{V}_V\right)\cdot {F}_{corr}\cdot {B}_{corr i}+{V}_V\cdot {C}_{RV}. $$

Solving Eq. 12 is a linear problem against the parameters *F*_*corr*_ and *V*_V_. For each basis function, *F*_*corr*_ and *V*_V_ in Eq. 12 are estimated by means of the linear least squares technique. *θ* is determined by searching the minimum sum of squared residuals between the estimated and observed data among all basis functions. From the determined *F*_*corr*_, *V*_V_ and *θ* values, *V*_*Tcorr*_ can be calculated. These computations are done for each voxel. In the present study, 50 logarithmically spaced precomputed basis functions with exponent values *θ* ranging from *λ* to 2 s^−1^ were used. PBF without TF correction *F* and PBF with TF and without blood volume correction *F*_*corr*'_ were also calculated using basis function method in the same way.

### Definition of lung fields

We defined the lung contralateral to the cancer-affected side as normal lung. We then defined the VOI on normal lung in the transmission images. The mean of the transmission values (linear attenuation coefficients) of the mediastinal blood pool and normal lung in 10 subjects were 0.1 and 0.03 (cm^−1^), respectively. We used an isocontour of 0.04 (cm^−1^) as a threshold. The resulting lung region corresponded to the lung field visually. Then, we manually removed the regions corresponding to large pulmonary vessels using PMOD^,^ version 3.4 (PMOD Technologies Ltd.). Within the remaining regions, we obtained the TF and the PBF with and without the TF.

### Data analysis

Intensity histograms of the TF and PBF were generated over the normal lung field for both the original and the TF-corrected data. From these histograms, we estimated the mean of the TF and PBF distribution. In addition, the kurtosis, a measure of the asymmetry of distribution, and the skewness, a measure of the peakedness of the distribution, were analyzed to evaluate the heterogeneity of the PBF among the different methods. We removed regions where the blood flow was higher than 6 mL/min/cm^3^ (more than 3 SD of PBF) from the calculation because these areas correspond to the pulmonary vasculature or the apical area affected by the spillover of the subclavicular vein.

We calculated the AIC in a voxel-wise manner and averaged the values over the normal lung for TF-corrected images with and without pulmonary blood volume correction.

### Statistical analysis

We statistically compared the mean, skewness, and kurtosis of the PBF with and without TF. We also compared the mean, skewness, kurtosis and AIC of the TF-corrected PBF with and without pulmonary blood correction. The statistical tests were performed using the Mann–Whitney *U* test with Matlab (MathWorks, Inc., Natick, MA).

## Results

The results of LAA_−950_ and FEV1/FVC were shown in Table 1. In seven of eight patients with smoking history, LAA_−950_ was less than 12% and emphysematous changes were confined to subpleural area. In one patient (No. 9), the emphysematous changes were severe and LAA_−950_ was as much as 25.9%. The results of FEV1/FVC were available in five patients. Three patients had normal, one had mild, and one had moderate airflow limitation.

**Fig. 2:**
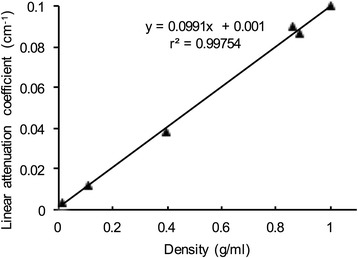
Correlation between the known density of various phantoms and the linear attenuation coefficient. The regression equation relating the two variables was a linear attenuation coefficient = 0.0991 (density) + 0.001; (*r* = 0.999)

The linear relationship between the linear attenuation coefficient and the known density for each phantom is shown in Fig. 2. The correlation coefficient *r* was 0.999. The intercept value (0.001 cm^−1^) was 1% of the value for water (0.1 cm^−1^) and was therefore ignored when calculating the calibration factor for converting the linear attenuation coefficient into a quantitative measure of density.

**Fig. 3:**
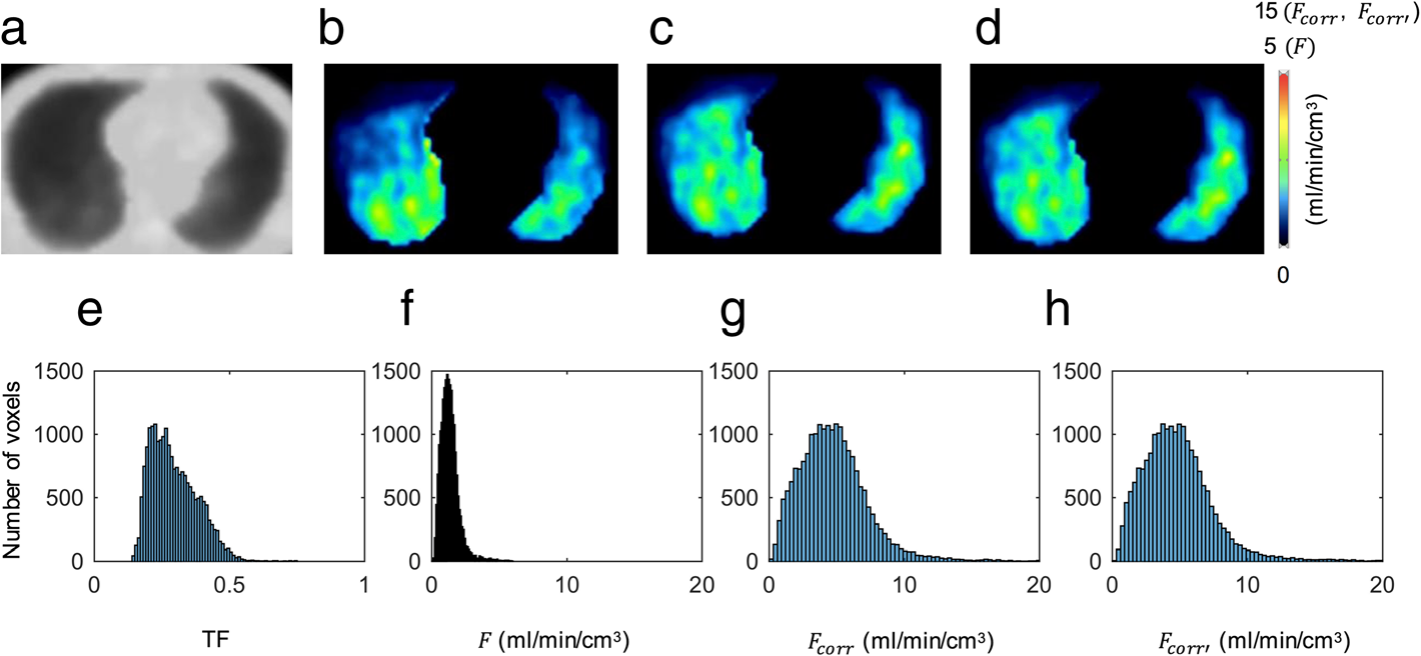
Transmission image (**a**), PBF image without TF correction (**b**), PBF image with TF and blood volume correction (**c**), PBF with TF and without blood volume correction (**d**), histograms of TF (**e**), PBF image without TF correction (**f**), PBF image with TF and blood volume correction (**g**), and PBF image with TF and without blood volume correction **(h**) in patient 1, who had right upper lobe lung cancer

Figure 3 shows a single slice obtained in a representative case illustrating the uncorrected PBF, the PBF corrected for both TF and blood volume, and the PBF corrected for TF but without blood volume correction. This patient had slight emphysematous change confined to subpleural area, but it was hard to detect corresponding PBF change. Visually, dorso-ventral gradient due to gravity in PBF appeared to be less prominent after TF correction. As for the TF-corrected PBF images, no visual differences were observed between the images with and those without blood volume correction. The histograms of PBF without TF correction, PBF with TF and without blood volume correction, and PBF with TF and blood volume correction are also shown in Fig. 3. After the TF correction, the skewed and peaked distributions of the PBF approached the Gaussian distributions, indicating that TF correction improved the heterogeneity of the PBF (Fig. 3 f, g, and h).

The quantitative results for PBF are presented in Table 2. The mean PBF after correction increased by 3.6 times compared with the uncorrected value. The skewness and the kurtosis of the TF-corrected PBF significantly decreased, compared with those of the uncorrected PBF, indicating that the distribution of the PBF had become more uniform and symmetrical. Since *V*_*V*_ was negligibly small (0.0005 ±0.0004), the TF-corrected PBF with and without blood volume correction did not differ significantly. The AIC also did not differ significantly between the TF-corrected images with and those without blood volume correction. The averaged *V*_*T*_ and ${V}_{T_{corr}}$ were 0.17 ± 0.03 and 0.60 ± 0.08, respectively.

**Table 2:** Summary of TF and PBF

	1 ‐ *V*_*air*_ (TF)	*F* (mL/min/cm^3^)	*F*_*corr*_ (mL/min/cm^3^)	${F}_{corr^{\hbox{'}}}$ (mL/min/cm^3^)
Mean	0.30±0.06	1.4±0.3	5.0±0.6^a^	5.3 ±0.6^a^
Skewness	0.6±0.3	1.2±0.5	0.9±0.4^a^	0.9±0.4^a^
Kurtosis	2.5±0.5	6.5±2.6	4.3±1.4^a^	4.6±1.6^a^

^a^The TF-corrected values differed significantly from those without TF correction (Mann–Whitney *U* test; *P* < 0.05)

**Fig. 4:**
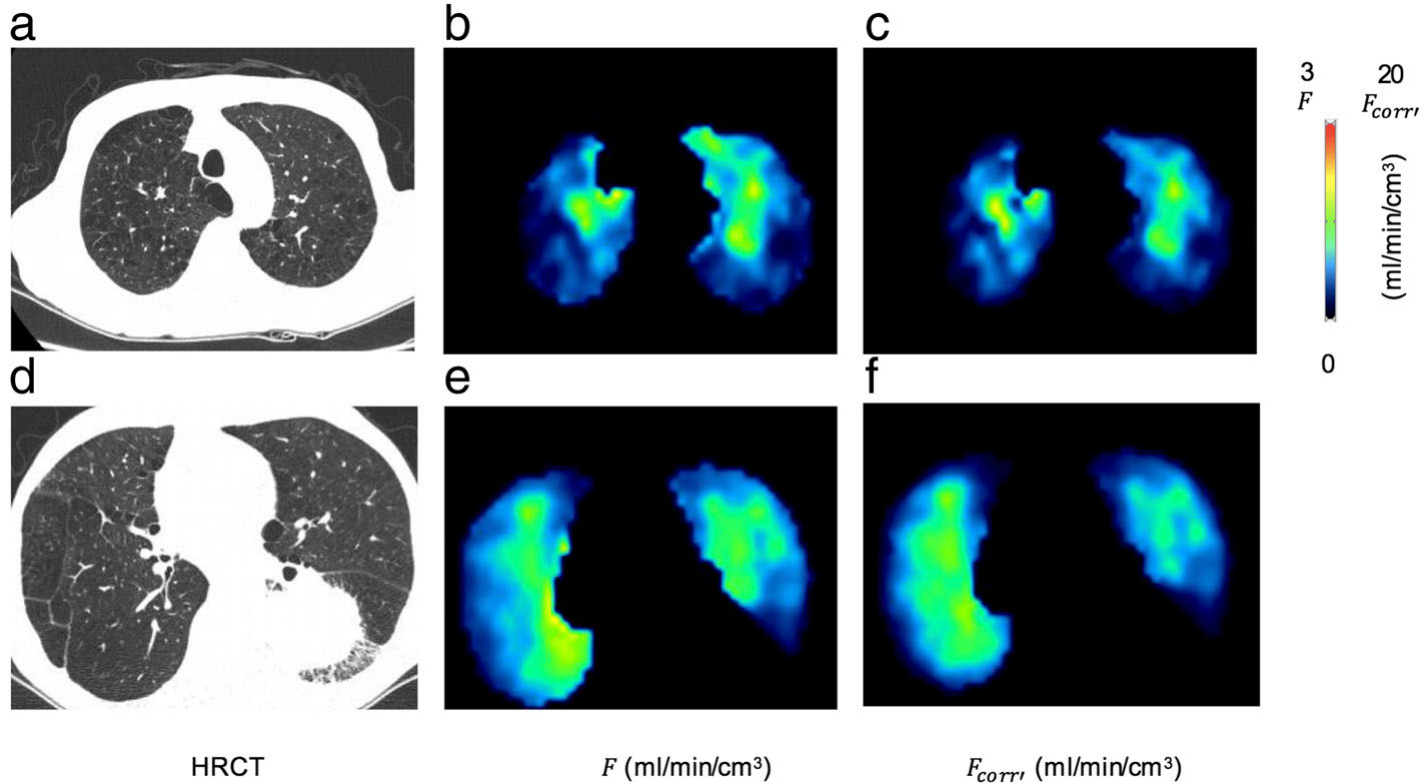
HRCT image (**a**), PBF image without TF correction (**b**), PBF image with TF and blood volume correction (**c**) of apical area and those of basal area (**d**-**f**) in patient 9, who had left lower lobe lung cancer and emphysematous changes

Figure 4 shows representative slices of the PBF and the PBF corrected for both TF and blood volume obtained in a case with severe emphysematous change. HRCT of corresponding slices were also shown. In Fig. 4b, c, low-PBF region remained after the TF correction, which corresponded to the area where emphysematous change was prominent in HRCT. On the contrary, in the relatively low PBF area in the right lung in Fig. 4e, PBF increased after the TF correction (Fig. 4f).

## Discussion

In the present study, we proposed a novel method for calculating the quantitative PBF, taking the TF and blood volume correction into account. We measured the TF using transmission scans obtained with a ^137^Cs external source. The averaged TF over the normal lung was 0.3, which was consistent with the result obtained from a ^68^Ga transmission scan [10, 11].

We measured the PBF of contralateral lung without TF correction by fitting the time activity curve of the lung to a one tissue compartment model. The averaged PBF without TF correction were 1.4 ± 0.3 mL/min/cm^3^ lung. Schuster et al. reported that the averaged PBF in normal 15 subjects was 1.4 ± 0.2 mL/min/cm^3^ lung using ^15^O-H_2_O PET (9). They obtained *F* and *V*_*T*_ separately by administering ^15^O-H_2_O twice, while we obtained both simultaneously using a single injection of ^15^O-H_2_O. Although our calculating method differed slightly from that of the above-mentioned previous report, the results were consistent.

Due to the history of smoking in eight of nine patients, we examined the presence of emphysematous changes in contralateral lung. Slight emphysematous changes confined to subpleural region were observed in seven of nine patients and the spirometry showed normal or mild airflow limitation. In one case, emphysematous changes were severe and there was moderate airflow limitation in spirometry. Pulmonary blood flow over whole lung is obtained by dividing cardiac output by functional residual capacity [9]. In the case with airflow limitation, functional residual capacity is expected to increase, which could reduce PBF. Actually, the averaged PBF was 0.9 mL/min/cm^3^ in the case with severe emphysema, which were lower than PBF of the remaining cases with slight or no emphysematous change (1.5 ± 0.3 mL/min/cm^3^).

We evaluated the effect of TF correction on quantitative PBF measurements. The ratio of PBF to the TF-corrected PBF was expected to be equivalent to the TF. The ratio of the average PBF to the TF-corrected average PBF was 0.3, which was reasonable since it corresponded to the mean TF (Table 2).

After TF correction, the PBF distribution became less skewed and less peaked. The asymmetrical distribution in the PBF without TF correction was thought to be mainly due to the vertical gradient in the PBF. A TF gradient in the dorso-ventral direction because of gravity was also reported in a ^68^Ga transmission study [10]. Therefore, the dorso-ventral gradient in the TF-corrected PBF, which is equivalent to the PBF divided by TF, was supposed to be smaller than that of the uncorrected PBF, reducing the asymmetry of the PBF distribution. Hopkins et al. evaluated the PBF and lung density in normal supine subjects using arterial spin labeling and proton density imaging during MRI examinations [18]. They reported that the dorso-ventral gradient in the PBF was reduced after the normalization of PBF according to density, consistent with our results.

In COPD or ILD, histological changes in the pulmonary vasculature have been reported [19–21]. The density distributions of the lung in these patients were heterogeneous. Since the raw PBF does not reflect the PBF per parenchyma, the PBF per lung parenchyma could be overestimated in low aeration areas and underestimated in high aeration areas. Thus, TF-corrected PBF images may contribute to clarifying the disease process, especially in these diseases. Actually, in one case with severe emphysematous change we found the region, where PBF was really decreased even after the TF correction, and the region where PBF per parenchyma was not so decreased after the TF correction.

We estimated the effect of the pulmonary blood volume on the calculation of PBF. *V*_*V*_ was negligibly small, and the model with blood volume correction was not superior to that without blood volume correction based on the AIC analysis. This result seems to contradict the volume fraction of the pulmonary vascular bed, which was 10–20%, derived from ^15^O-CO PET [10]. The pulmonary vascular bed is composed of arterial (precapillary), capillary, and venous (postcapillary) components. In our formulation, *V*_*V*_ corresponds to precapillary blood volume fraction [22] because first-pass extraction of ^15^O-H_2_O by lung tissue could be considered to be 100% [8]. The precapillary component of lung could be estimated to be about 0.03 per lung volume based on a histological analysis [23]. We could not distinguish such small contributions from the precapillary blood volume in the PET data. Although the contribution of pulmonary vascular bed was not taken into account in the former studies of measuring PBF with ^15^O-H_2_O PET [8, 9], the results were in good agreement with the PBF derived from microsphere measurements, which supported our result.

The present study had several limitations. First, the types of PET scanners currently available are limited. Although we used a PET scanner that was equipped with an external transmission source, PET/CT scanners with CT-based transmission scans are mainstream among PET devices. CT images could be used to derive the TF [2, 3] instead of transmission images; however, careful registration of the CT images with the PET images is required because of respiratory movement. TF derived from CT taken at deep respiration may induce some errors caused by the misregistration with the PET emission data. Therefore, CT at shallow breathing [2] or Cine-CT over complete breathing period [3] could be useful. Second, we could not divide the capillary and post-capillary components using the present TF correction method. In our definition of TF, true lung parenchyma components (alveoli, bronchioles, and interstitium) in addition to capillary and post-capillary vascular components were considered as “parenchyma”. To obtain the PBF per true parenchyma, information on the capillary and post-capillary vascular components, which could be derived from ^15^O-CO PET, is required. Rhodes et al. [10] estimated the volume fraction of the true lung parenchyma components by subtracting the ^15^O-CO vascular component image from the density image derived from the transmission scan. Third, we did not consider the gradient-dependent difference in the TF that exists between the lung parenchyma and the blood component precisely. There was a vertical gradient in the volume fraction of the true parenchyma (0.12 at the ventral portion, and 0.16 at the dorsal portion), while the volume fraction of the blood had a steeper gradient (0.075 at the ventral portion and 0.21 at the dorsal portion). Therefore, if we use the TF of the true parenchyma in the PBF correction, the dorso-ventral gradient in the corrected PBF might be larger than our present results. In other words, we might have overcorrected the vertical heterogeneity of the PBF.

## Conclusions

We have developed a novel method of calculating the TF-corrected PBF using ^15^O-H_2_O PET. We derived the TF from transmission images. We applied this method to the contralateral lung in patients with lung cancer. After TF correction, the heterogeneous distribution of the PBF arising from gravity became more uniform.

## Appendix

### Abbreviations

*ρ*_*lung*_ = average density of lung over voxel (g/cm^3^)

*ρ*_*air*_ = density of air (g/cm^3^).

*ρ*_*pa*_ = density of lung parenchyma (g/cm^3^).

*ρ*_*v*_ = density of blood (g/cm^3^).

*V*_*air*_ = fraction of unit volume occupied by air at a specific voxel.

*V*_*pa*_ = fraction of unit volume occupied by parenchyma at a specific voxel.

*V*_*V*_ = fraction of unit volume occupied by blood at a specific voxel.

*T*_*lung*_ = linear attenuation coefficient of lung (cm^−1^).

*T*_*med*_ = linear attenuation coefficient of mediastinum (cm^−1^).

*F* = PBF per lung volume (mL/s/cm^3^).

*V*_*T*_ = distribution volume of water.

*λ* = physical decay constant of ^15^O (0.00567 s^−1^).

*F*_*corr*_ = PBF per lung parenchyma(mL/s/cm^3^).

${V}_{T_{corr}}$ = distribution volume of water in lung parenchyma.

*F*_*corr*’_ = PBF per lung parenchyma without pulmonary circulation blood volume correction (mL/s/cm^3^).

${V}_{T_{corr}'}$ = distribution volume of water in lung parenchyma without pulmonary circulation blood volume correction.

## Abbreviations

AIC: Akaike information criteria
COPD: Chronic obstructive pulmonary disease
CT: Computed tomography
DRAMA: Dynamic row-action maximum-likelihood algorithm
FDG: Fluorodeoxyglucose
FEV1/FVC: the ratio of forced expiratory volume in 1 s to forced vital capacity
FWHM: Full width at half maximum
HR: High resolution
HU: Hounsfield unit
ILD: Interstitial lung disease
LAA_−950_: Volume fraction of lung parenchyma with attenuation coefficient of less than −950 HU to the whole contralateral lung
PBF: Pulmonary blood flow
PET: Positron emission tomography
SD: Standard deviation
SUV: Standardized uptake value
TF: Tissue fraction
VOI: Volume of interest
